# Eosinophilic arthritis—A case report

**DOI:** 10.1002/ccr3.3737

**Published:** 2021-01-05

**Authors:** Hideaki Yamabe, Fumiko Kudo, Michiko Shimada

**Affiliations:** ^1^ Internal Medicine Masuda Hospital Goshogawara Japan; ^2^ Department of Nephrology Hirosaki University Hospital Hirosaki Japan

**Keywords:** arthritis, corticosteroids, eosinophilia, eosinophilic arthritis, nonsteroidal anti‐inflammatory drugs

## Abstract

Eosinophilic arthritis is characterized by arthritis, eosinophilia, normal laboratory findings, unresponsiveness to nonsteroidal anti‐inflammatory drugs and favorable response to corticosteroids. We diagnosed a female patient with this rare disease.

## Graphical abstract

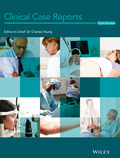

## 1 INTRODUCTION

Eosinophilic arthritis is characterized by arthritis, eosinophilia, normal laboratory findings, and unresponsiveness to nonsteroidal anti‐inflammatory drugs. We diagnosed a female patient with eosinophilic arthritis and treated her using a corticosteroid. Her symptoms were relieved after several days. It is important for physicians to be aware of this rare disease.

Eosinophils are granulocytes derived from progenitor cells of monocytes, macrophages, and basophils. They are components of the innate immune system, and eosinophils have various functions, including defense against parasites and intracellular bacteria and modulation of immediate hypersensitivity reactions.^1^ The normal peripheral blood eosinophil count is variable; however, a count of > 500 cells/mm^3^ is considered as elevated. Peripheral eosinophilia is characterized as follows: mild, 500‐1500 cells/mm^3^; moderate, 1500‐5000 cells/mm^3^; and severe, >5000 cells/mm^3^. The common causes of eosinophilia include allergic or atopic disorders and parasitic infections. In our case, the patient complained of edema and pain in her wrists and ankles and presented moderate eosinophilia. However, she had normal laboratory findings and was unresponsive to nonsteroidal anti‐inflammatory drugs (NSAIDs). Therefore, we diagnosed her with eosinophilic arthritis, which is quite rare.

## 2 CASE REPORT

**FIGURE 1:**
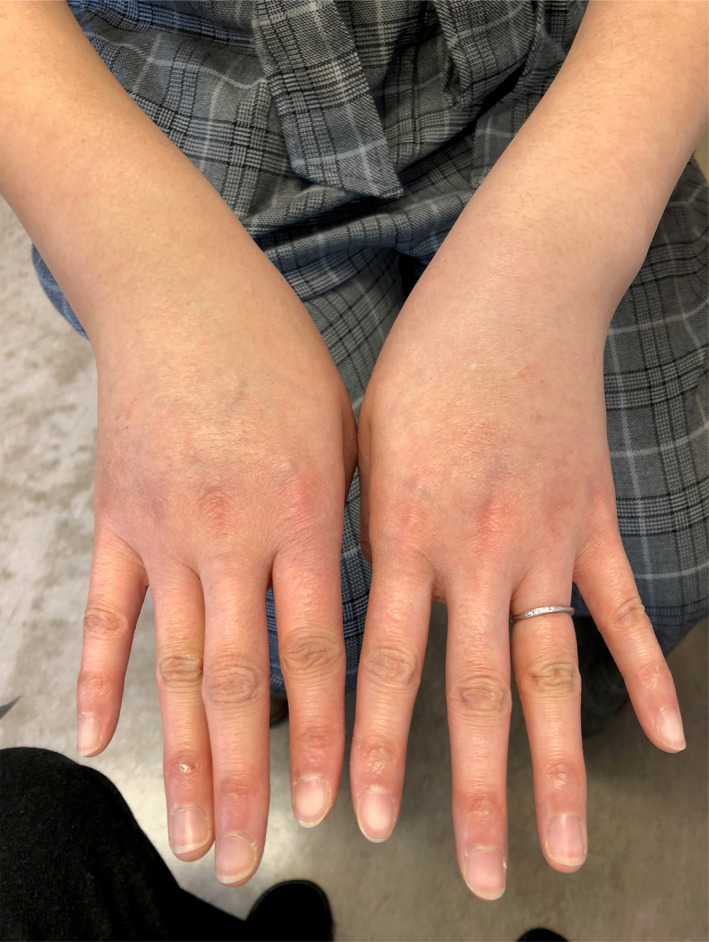
Edema in both the wrists was observed, which was more severe on the right side

**FIGURE 2:**
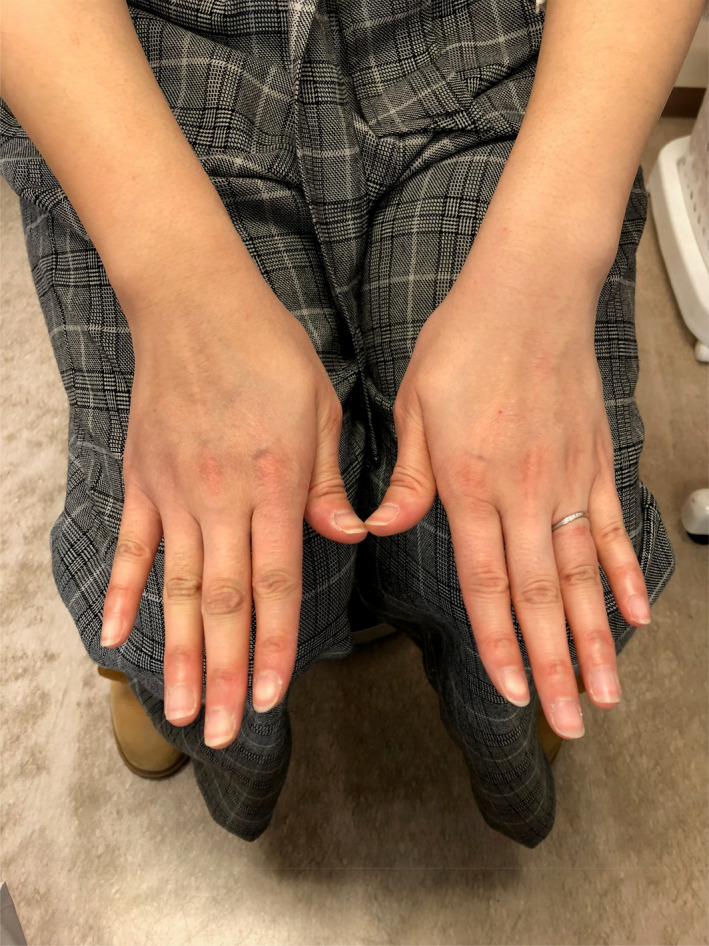
Edema in the wrists was disappeared

A 30‐year‐old Japanese woman presented to an orthopedist after experiencing edema and pain on her wrists and ankles for a week. She was prescribed with NSAID, which did not relieve her symptoms. This resulted in her visiting our hospital. She had been healthy and had not used any ordinary drug or supplement. Additionally, she had no history of allergies and parasitosis. However, her mother suffered from hyperthyroidism according to her family history. A physical examination confirmed edema in both her wrists and ankles, with the edema being more severe on the right side (Figure 1). Pitting edema of the lower limbs, goiter, cardiac murmur, and abnormal lung sounds were not detected. Her blood pressure was 110/67 mmHg, and her chest X‐ray and pulmonary function results were normal. Her peripheral blood cell counts were as follows: white blood cells (WBCs), 11 400 cells/mm^3^; erythrocytes, 4.99 million cells/mm^3^; and platelets, 253 000 cells/mm^3^. Her hemoglobin was 13.6 g/dL, and hematocrit was 41.7%. The eosinophil level was 15.5% (1767 cells/mm^3^). Her blood biochemical analysis results were as follows: total protein, 7.6 g/dL; serum albumin, 4.5 g/dL; Na, 138 mEq/L; K, 4.3 mEq/L; Cl, 104 mEq/L; serum creatinine, 0.66 mg/dL; estimated glomerular filtration rate, 85.1 mL/min; aspartate aminotransferase, 20 IU/L; alanine transaminase, 20 IU/L; blood sugar, 104 mg/dL; thyroid‐stimulating hormone, 1.23 μU/mL; free triiodothyroxine, 2.63 pg/mL; free thyroxine, 0.94 ng/dL; and one‐hour erythrocyte sedimentation, 8 mm. An immunological examination revealed normal levels of rheumatoid factor, antistreptolysin O, antistreptokinase, anticyclic citrullinated peptide antibodies, antideoxyribonucleic antibodies, antiribonucleic acid antibodies, perinuclear antineutrophil cytoplasmic antibodies, cytoplasmic antineutrophil cytoplasmic antibodies, and immunoglobulin E. We treated her with a NSAID but her symptoms were not relieved. Her WBC and eosinophil levels increased to 11 900 cells/mm^3^ and 19.4% (2308 cells/mm^3^), respectively. Therefore, we diagnosed her with eosinophilic arthritis and treated her with prednisolone (30 mg/day). Within several days, her edema and pain were relieved (Figure 2), and her eosinophil levels decreased to 0.2% (25 cells/mm^3^). Prednisolone was gradually tapered and ceased two months later, no occurrence of relapse for at least one year.

## 3 DISCUSSION

We report the rare case of a 30‐year‐old Japanese woman with eosinophilic arthritis. Polyarthritis with peripheral blood eosinophilia is associated with several diseases. Peripheral blood eosinophilia is common among patients with concomitant allergies or helminthic infections.^2^, ^3^ Our patient did not have allergies or helminthic infections. Differential diagnosis considered for the patient with arthritis and eosinophilia was summarized.^4^ It has been reported that some drugs cause drug‐induced hypersensitivity syndrome with eosinophilia and arthritis.^5^ However, our patient did not take any medicine before experiencing symptoms of eosinphilic arthritis. Rheumatoid arthritis rarely shows persistent eosinophilia.^6^ However, our patient did not show any laboratory findings suggestive of rheumatoid arthritis. Hypereosinophilic syndrome (HES), first described by Hardy and Anderson in 1968,^7^ is well known and the most probable diagnosis for our patient. It is characterized by peripheral blood eosinophilia and polyarthritis with organ system involvement or dysfunction associated with eosinophilia in the absence of parasitic, allergic, or other secondary causes. However, our patient did not show any organ involvement and abnormal laboratory findings. Tay first reported eosinophilic arthritis in 1999, which is a distinct condition similar to HES.^8^ Tay described 10 patients presenting with polyarthritis and eosinophilia. None had constitutional symptoms or abnormal laboratory findings except high eosinophil counts. NSAIDs did not relieve the joint symptoms. However, oral corticosteroids administration resulted in rapid and favorable efficacy against arthritis in six of eight patients. Therefore, we diagnosed our patient with eosinophilic arthritis as she had moderate eosinophilia (>1500 cells/mm^3^), normal laboratory findings, unresponsiveness to NSAIDs, and favorable response to corticosteroids. The pathogenesis of this disease was unknown and could have resulted from a reaction to some unknown agents or allergens.

## 4 CONCLUSION

We report the case of a 30‐year‐old Japanese woman presenting with edema and pain in her wrists and ankles, who was diagnosed with eosinophilic arthritis. The etiology of this disease is unknown, and it is important for physicians to be aware of this rare disease.

## CONFLICT OF INTEREST

None declared.

## AUTHOR CONTRIBUTIONS

HY and FK: treated the patient and described the manuscript. MS: revised the manuscript.

## STATEMENT OF ETHICS

Written informed consent was obtained from the patient for publication of this case report and any accompanying images. A copy of the written consent is available for review by the Editor‐in‐Chief of this journal.

## Data Availability

All data that support the findings of this study are included in this case report. Details are available on request from the corresponding author.
